# Biomarkers for autism spectrum disorder: opportunities for magnetoencephalography (MEG)

**DOI:** 10.1186/s11689-021-09385-y

**Published:** 2021-09-15

**Authors:** Timothy P. L. Roberts, Emily S. Kuschner, J. Christopher Edgar

**Affiliations:** Dept. of Radiology, Lurie Family Foundations MEG Imaging Center, Children’s Hospital of Philadelphia, 3401 Civic Center Blvd., Philadelphia, PA 19104 USA

## Abstract

This paper reviews a candidate biomarker for ASD, the M50 auditory evoked response component, detected by magnetoencephalography (MEG) and presents a position on the roles and opportunities for such a biomarker, as well as converging evidence from allied imaging techniques (magnetic resonance imaging, MRI and spectroscopy, MRS). Data is presented on prolonged M50 latencies in ASD as well as extension to include children with ASD with significant language and cognitive impairments in whom M50 latency delays are exacerbated. Modeling of the M50 latency by consideration of the properties of auditory pathway white matter is shown to be successful in typical development but challenged by heterogeneity in ASD; this, however, is capitalized upon to identify a distinct subpopulation of children with ASD whose M50 latencies lie well outside the range of values predictable from the typically developing model. Interestingly, this subpopulation is characterized by low levels of the inhibitory neurotransmitter GABA. Following from this, we discuss a potential use of the M50 latency in indicating “target engagement” acutely with administration of a GABA-B agonist, potentially distinguishing “responders” from “non-responders” with the implication of optimizing inclusion for clinical trials of such agents. Implications for future application, including potential evaluation of infants with genetic risk factors, are discussed. As such, the broad scope of potential of a representative candidate biological marker, the M50 latency, is introduced along with potential future applications.

This paper outlines a strategy for understanding brain dysfunction in individuals with intellectual and developmental disabilities (IDD). It is proposed that a multimodal approach (collection of brain structure, chemistry, and neuronal functional data) will identify IDD subpopulations who share a common disease pathway, and thus identify individuals with IDD who might ultimately benefit from specific treatments. After briefly demonstrating the need and potential for scope, examples from studies examining brain function and structure in children with autism spectrum disorder (ASD) illustrate how measures of brain neuronal function (from magnetoencephalography, MEG), brain structure (from magnetic resonance imaging, MRI, especially diffusion MRI), and brain chemistry (MR spectroscopy) can help us better understand the heterogeneity in ASD and form the basis of multivariate biological markers (biomarkers) useable to define clinical subpopulations. Similar approaches can be applied to understand brain dysfunction in neurodevelopmental disorders (NDD) in general. In large part, this paper represents our endeavors as part of the CHOP/Penn NICHD-funded intellectual and developmental disabilities research center (IDDRC) over the past decade.

## Background

Although the term “biomarker” (or “biological marker”) often brings to mind a blood test or genetic screen, laboratories around the world are working to identify structural brain imaging measures (such as diffusion MRI) and functional brain measures (such as electrophysiological measures from electroencephalography, EEG, or magnetoencephalography, MEG) as biomarkers for clinical disorders where there is currently no biomarker. For example, there are large research programs seeking brain markers for schizophrenia (e.g., [1–3]) and autism spectrum disorder (ASD) (e.g., [4–6]). Whereas these studies have traditionally examined a single brain measure (e.g., cortical thickness, cerebral blood flow or neural activity), mapping regional differences in both brain structure and function (and their relationships) might be expected to better account for varied behavioral phenotypes (in contradistinction to blood-based chemical biomarker assays); such studies are now possible given the spatial resolution of modern brain imaging as well as advanced analysis approaches (e.g., [7–11]).

Brain biomarkers have a variety of potential clinical uses. For example, biomarkers may provide diagnostic information and help predict outcome, as well as identify subpopulations within a clinically diagnosed disorder who share a common disease pathway. With respect to patient treatment and clinical trials, biomarkers may also provide a basis for participant enrollment enrichment (i.e., as inclusion criteria) as well as being employed as early signals of efficacy via acute evidence of biological activity (interpreted as target engagement). Ultimately biomarkers might be employed to direct an individual patient to an optimal, or even personalized, treatment regimen.

After more than 40 years of brain imaging research, a key finding is the very significant heterogeneity in brain measures in most neuropsychiatric disorders, especially when considered within the context of a specific DSM diagnosis. Indeed, brain studies comparing controls and DSM patient groups most often observe biomarkers with small to medium effects (frequently represented via Cohen’s *d*’), and thus with substantial overlap between control and patient groups on the biomarker assay (e.g., [10, 12, 13]). Inherently, such overlap implies that many biomarkers will have poor sensitivity and specificity as discriminative clinical *diagnostic* markers.

Such *diagnostic* heterogeneity has suggested an alternative approach, namely in the use of biomarkers for subpopulation stratification [14], as well as in the adoption and use of biomarkers crossing DSM diagnoses. This is exemplified in the Research Domain Criteria (RDoC) initiative [15–17], with NIMH Director Josh Gordon recently noting that, “More and more, scientists and clinicians alike began to appreciate the blurred boundaries between categorical disorders, and the heterogeneity within them. Furthermore, results of studies aimed at examining the neural underpinnings of disorders defined by traditional diagnostic criteria frequently failed to replicate. Taken together, these findings led to the recognition that traditional diagnostic systems were not capturing the true underlying structures of mental illnesses” [18].

A cross-diagnostic approach is intrinsic to the IDD diagnosis, given the overlap of domains and systems impacted, as well as varying etiologies, including genetic disorders, metabolic syndromes, and non-syndromic/non-specific presentations. IDD diagnosis globally represents altered developmental trajectories, present from birth, in physical, developmental, and emotional functioning. Multiple body parts and systems (e.g., sensory or nervous system, metabolism) are often affected. An intellectual disability (ID) diagnosis, made in conjunction with identified etiology, specifically reflects impairments in intellectual and adaptive functioning (standardized scores generally < 70) with childhood onset. Also of note is that given possible (and perhaps even likely) control and patient group differences in brain maturation, such age-related changes may temporally constrain the use of brain biomarkers as diagnostic markers (for an extended discussion, see [19]).

Also of note is that individuals with IDD often have co-occurring conditions. Indeed, the NICHD Intellectual and Developmental Disabilities Research Centers (IDDRC) 2020 FOA notes that, “Individuals with IDD experience behavioral symptoms and mental health conditions at considerably higher rates than the general population, including behavioral symptoms such as depression, aggression, or suicidal ideation or mental health conditions such as attention deficit hyperactivity disorder (ADHD), bipolar disorder, or psychotic disorders. These can be extremely challenging to manage in individuals with IDD due to the language, cognitive, and sensory impairments that often impede traditional strategies for evaluation and treatment” [20]. IDDRC research accommodates diversity within the IDD diagnosis; for example, with respect to assessment research, an IDDRC goal is “Development of a biomarker, assessment measure, or clinical intervention for more than one IDD condition or a group of related IDD conditions that share a common feature or metabolic or molecular pathway.” With respect to outcome measures, a key IDDRC goal is “Development of a measure or biomarker that can be applied to more than one IDD conditions that share a common feature or metabolic or molecular pathway.”

Within our group, a focus has been attempting to understand the biology that is associated with auditory neural encoding processes in pediatric populations, with and without neurodevelopmental disorders. As detailed in the following section, a primary finding is that the latencies of auditory cortex evoked responses, detected by MEG and measured in milliseconds, are often delayed in children with ASD. Cross-diagnostic research in our laboratory has shown that auditory encoding response delays are also observed in genetic conditions that put one at risk for ASD, even in the absence of conclusive symptomatology of ASD (such as 16p11.2 deletion syndrome and 47, XYY syndrome). In another line of research, multimodal imaging (MEG+MRI+MRS) has suggested delineation of an ASD subpopulation sharing a common disease pathway. As findings are reviewed, we also outline several promising and necessary future directions for research, with a focus on the need to include more severely impaired children than those typically offered the opportunity to participate in brain imaging research, thereby increasing the generalizability of findings and ultimately access to biomarker technology and pursuant candidate therapies (e.g., [21]).

## Use of multimodal imaging to understanding auditory encoding abnormalities in children with autism spectrum disorder (ASD)

### Auditory encoding neural processes

One promising biomarker candidate for ASD is a measure of how fast auditory information can be encoded. In the time domain, the auditory M50 response (EEG = P50 or P1) and the auditory M100 response (EEG = N100 or N1) are often examined. In our laboratory, we assess auditory encoding processes in left and right primary/secondary auditory cortex, the primary generators of the M50 and M100 responses [22–26]. Left and right auditory cortex activity needs to be separately examined given an extensive literature demonstrating hemispheric differences in auditory cortex maturation rates [27–32], and given many studies showing that ASD and TDC group differences are hemisphere specific [28, 33–37]. And although in older children the auditory M100 response is of interest, in younger children (< ~ 10 years old) the M100 response is often not fully developed and may not be readily observed [28, 38–40]. For these reasons, in younger children, M50 is generally a preferred measure to access auditory cortex response, and much of the ensuing discussion focuses on this robustly determined component in children younger than 10 years.

### Delayed cortical auditory encoding in autism spectrum disorder

**Fig. 1:**
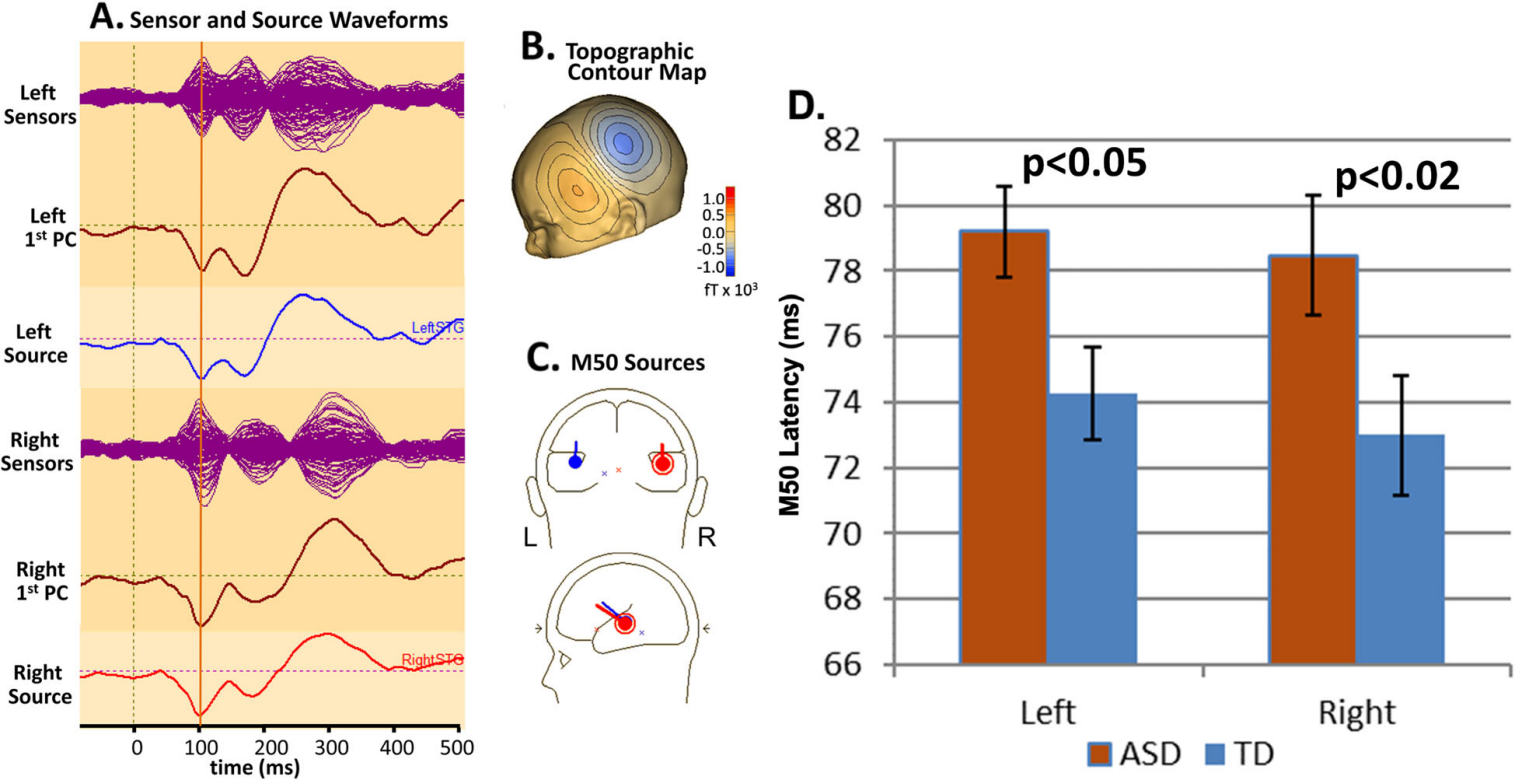
**A** Auditory evoked responses depicted in sensor and source space identify the M50 (red vertical line), **B** confirms the magnetic field topography (i.e., left hemisphere M50 magnetic field topography) and **C** the two-dipole source model used to extract the source waveforms in **A** and **D** shows group level M50 latency differences (mean ± SEM), significant bilaterally and with ~ 5 ms M50 latency delay in ASD compared to TDC

Our studies, primarily in school-aged children (6–15 years) and conducted in a conventional CTF-275 biomagnetometer, have identified M50 (as well as the later M100) latency delays in ASD [28, 33, 36, 41] (see Fig. 1). Sample sizes of children with ASD in these reports range from 25 in the earlier studies to over 100 in more recent reports. These findings have been reproduced in other laboratories and point to atypical auditory cortex neural activity in ASD (e.g., [37, 42, 43] and also see recent meta-analysis [44]). Most studies involve simple sinusoidal tone stimuli of typically 300 ms duration (in our studies with 10 ms onset/offset ramps) and frequency in the range 200 Hz to 1 kHz. Finding direct associations between atypical auditory cortex activity and behavioral/clinical measures has been elusive, likely due to the known heterogeneity within ASD, as well as the fact that our existing studies have primarily focused on children with ASD exhibiting generally mild language and cognitive impairment. However, in a recent study that included more severely impaired children with ASD (see below), associations between M50 latency and both language ability and general cognitive ability (non-verbal IQ) were observed [45].

With respect to the diagnostic specificity of M50 latency delays, our laboratory has found delayed auditory cortex responses among individuals with genetic syndromes associated with a higher than typical incidence of ASD, namely 16p11.2 deletion syndrome (*N* = 137) and 47, XYY syndrome (*N* = 120) [46, 47], leading to the hypothesis that M50 latency delays will be observed in many children with IDD, with this “lack of specificity” suggesting a *common biological pathway* across etiologies and diagnoses.

### Auditory encoding in children with ASD with cognitive and language impairment

**Fig. 2:**
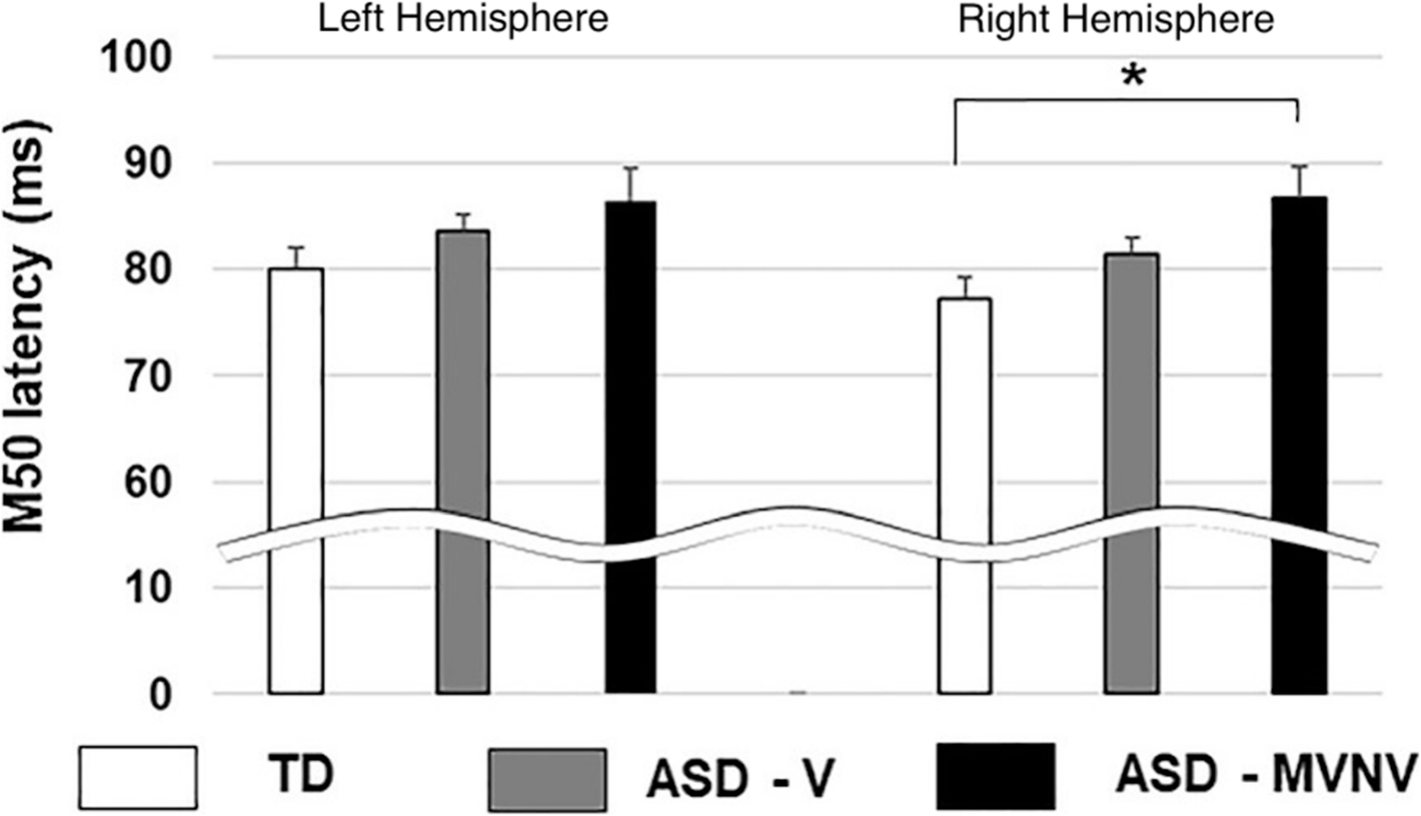
Left and right M50 response latencies are delayed in verbal children with ASD (ASD-V) and further delayed in minimally verbal/non-verbal children with ASD (ASD-MVNV)

In a preliminary study [45], the MEG-PLAN approach (discussed below) allowed for observation of M50 in a wider range of children with ASD, confirming delayed M50 responses in verbal children with ASD (ASD-V) but also extending M50 latency findings to a cohort of 16 minimally verbal/non-verbal (ASD-MVNV) children. In particular, M50 responses were much more delayed in ASD-MVNV than ASD-V (a mean delay of 8 ms in ASD-MVNV versus TD controls and 4 ms in ASD-MVNV versus ASD-V), and with M50 delays more prominent in the right compared to left hemisphere (Fig. 2). The ASD-MVNV findings demonstrate the feasibility of conducting advanced imaging research in this often understudied population. Results show a large group-difference effect in ASD-MVNV, likely related to the degree of language and cognitive impairment in these children. Findings also suggest that the M50 latency may have a continuous, dimensional role across a range of cognitive/language abilities in the full autism spectrum.

### M50 latency delays in NVIQ-matched IDD children without ASD

Further insight into the above was gained via a feasibility study in children with ASD-MVNV as well as IDD [48]. A clinical control group of 10 children with IDD (mixed etiology) *also showed prolonged M50 latency* compared to ASD-MVNV group described above (with no group difference in NVIQ, IDD = 59 ± 4 vs. ASD-MVNV = 57 ± 2, *p* > 0.05). These prolonged latencies were evident despite less impaired language/communication ability (VABS-CD: IDD = 63 ± 5 versus ASD-MVNV = 47 ± 3, *p* < 0.01; PPVT-4: IDD = 58 ± 5 versus ASD-MVNV = 33 ± 2, *p* < 0.001), confirming M50 latency as a cross-diagnostic measure sensitive to general cognitive impairment.

In summary, our MEG findings demonstrate a delay in the M50 auditory response in ASD that is exacerbated in MVNV-ASD and that may also be delayed in IDD-clinical controls. This commonality, rather than being considered a lack of specificity, could be viewed as a common signature of shared pathophysiology, and potentially as a basis for treatment stratification. The sections below describe our work to better understand the biology implementing typical and atypical auditory encoding.

### Multimodal underpinnings of the M50 response: identification of subpopulations

Two imaging measures have offered insight into the brain physiology associated with M50 latency: diffusion MRI (dMRI) and spectrally edited magnetic resonance spectroscopy (MRS). We have used these measures to explain variance in the M50 latency of TD children, and to identify extreme outliers to this explanation in a subpopulation of children with ASD, implying alternative and/or additional biological factor(s). The search for a biological basis of a delayed cortical response facilitates identification of ASD subpopulations based on biological rather than clinical criteria (i.e., in general, mirroring adoption of an RDoC research strategy [15, 17]).

**Fig. 3:**
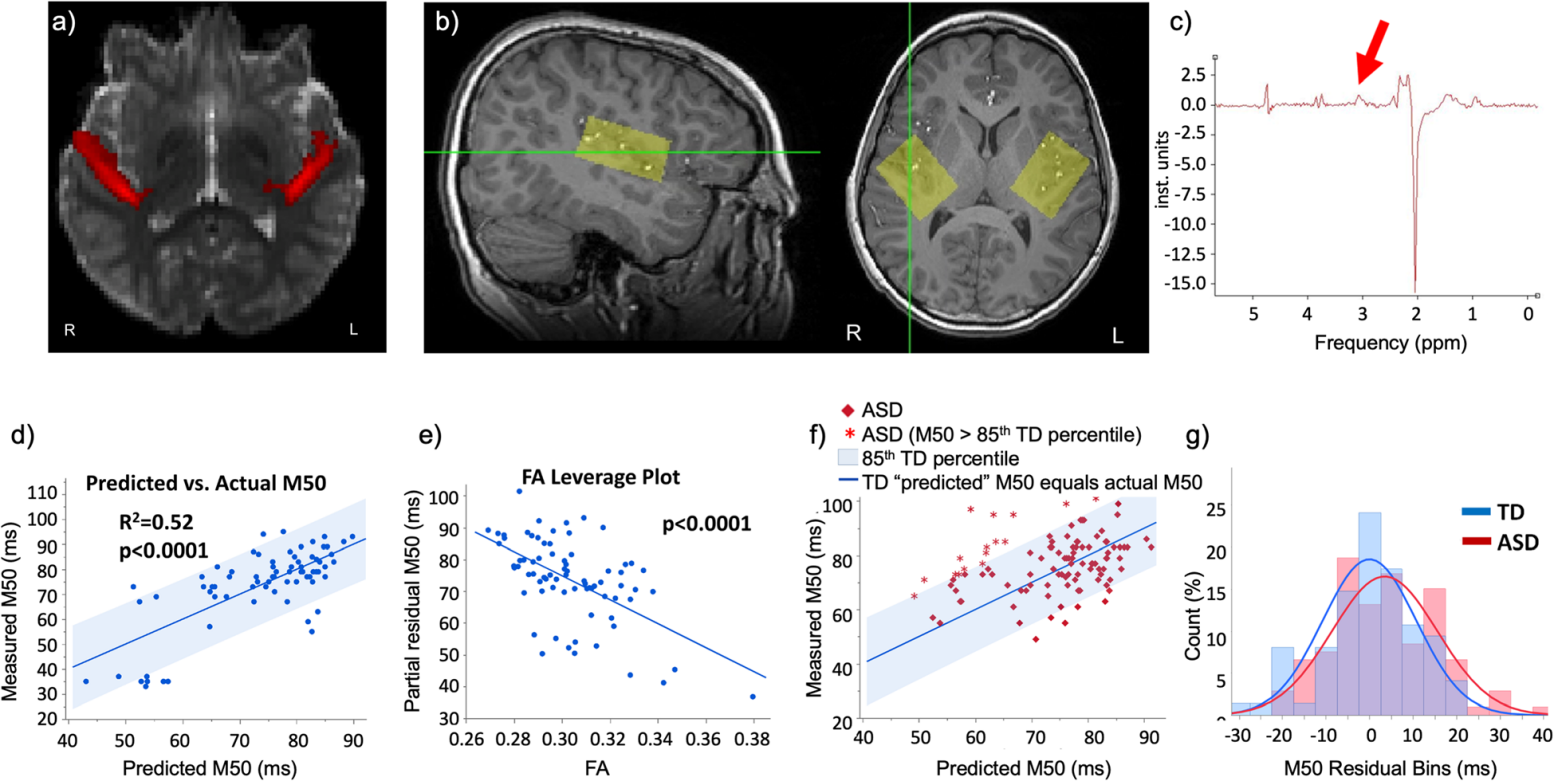
Multimodal approaches to mechanism and statistical definition of ASD subpopulations: **a** white-matter fiber tracking of the thalamocortical auditory radiations, defined using high angular resolution diffusion imaging (HARDI). **b** Sagittal and axial depiction of the voxel placement for spectrally edited MEGAPRESS MRS, yielding GABA estimates (**c**). Modeling M50 latency (from Fig. 1) using the acoustic radiations FA along with age in **d** allowed prediction of M50 latency in TD children (accounting for 52% of the variance) with **e** white-matter FA a significant predictor of M50 latency (*p* < 0.0001). The model, although still significant (but with different coefficients), did not perform as well in the ASD cohort, likely due to the heterogeneity of the ASD cohort. However, and in fact addressing the heterogeneity of ASD (**f**), a subpopulation of extreme M50 latency outliers was identified, as depicted in the histograms of residual latency (deviation from the TD model). This subpopulation was characterized by having significantly lower GABA than the ASD children that conformed to the TD model [1]

In Roberts et al. [49], a relationship between auditory evoked M50 latency and thalamocortical auditory radiation fractional anisotropy (FA) measured by dMRI was observed in TDs but was less evident across a large (*n* = ~ 100) ASD population (Fig. 3). In this study, conformity (or, specifically, lack thereof) to a biophysical model (derived from TD controls) defined an “outlier” ASD population in a multivariate fashion (i.e., not simply *long* M50 latencies, but “longer than would be predicted based upon thalamocortical FA values”), with the suggestion that such identification of biologically distinct subgroups would be needed to identify individuals most likely to benefit from a specific treatment targeting a specific brain abnormality [21, 50]. And, of note, this subpopulation of extreme M50 latency outliers (depicted in the histograms of residual latency (deviation from the TD model)) was characterized by having significantly lower GABA than the ASD children that conformed to the TD model. Extension of these findings to more significantly impaired children (see following section) is now needed to firmly establish such subgroups.

## Obtaining brain imaging measures in lower-functioning children

It is unknown if the multimodal findings discussed in the section “Use of multimodal imaging to understanding auditory encoding abnormalities in children with autism spectrum disorder (ASD)” are valid and generalizable across the entire autism spectrum. As alluded to in the sections “Auditory encoding in children with ASD with cognitive and language impairment” and “M50 latency delays in NVIQ-matched IDD children without ASD,” to obtain MEG measures in lower-ability children, it was necessary to develop a strategy: MEG-PLAN (MEG Protocol for Low-Language/Cognitive Ability Neuroimaging) [51], to allow successful evaluation of the above-described brain measures in MVNV-ASD. Inclusion of more significantly impaired children in brain imaging research is critical not only from a scientific viewpoint (for generalizability) but also from a societal standpoint (permitting broader access to research, intrinsic to philosophies of inclusivity).

**Fig. 4:**
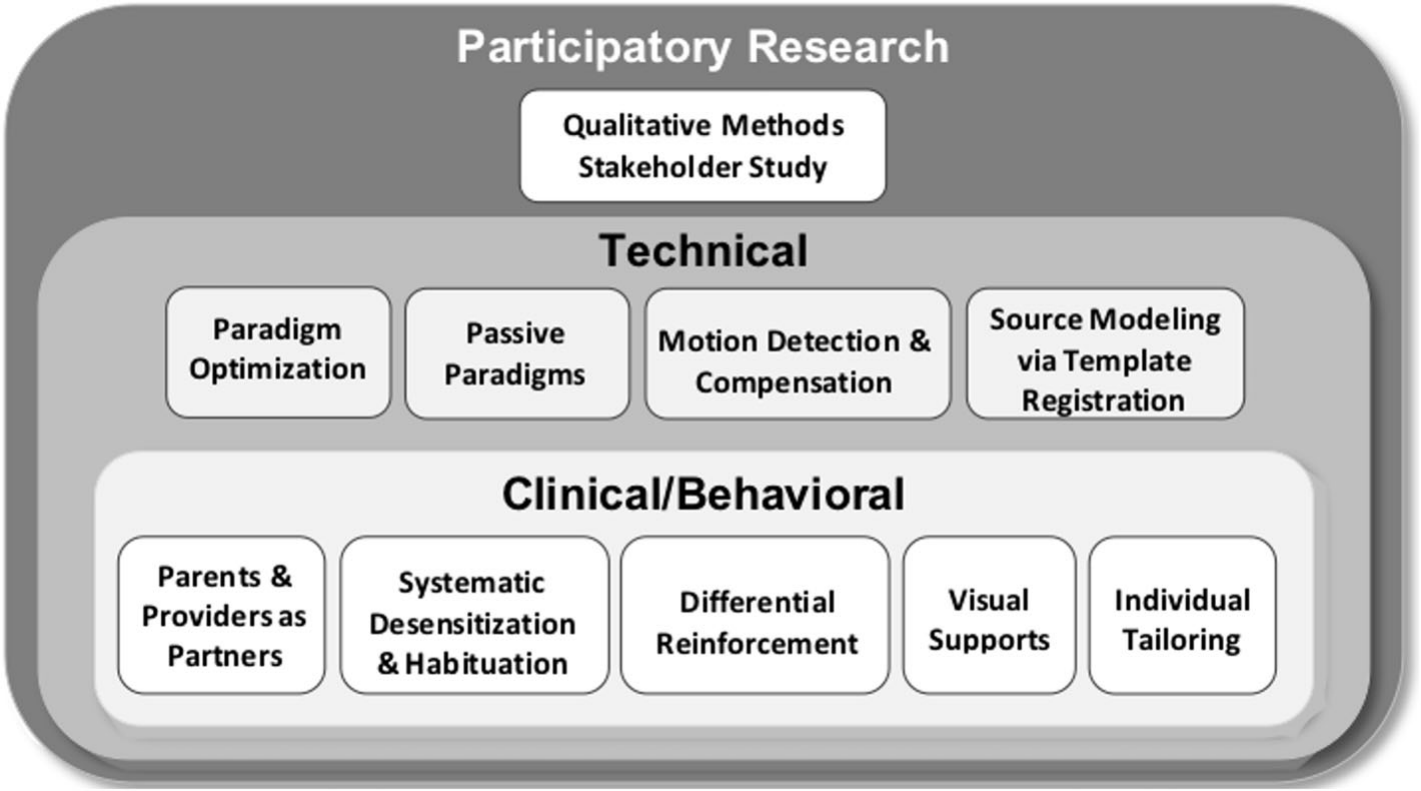
Schematic of the MEG Protocol for Low-Language/Cognitive Ability Neuroimaging (MEG-PLAN)

MEG-PLAN integrates clinical and technical supports to personalize the scan experience, maximize tolerability, and optimize data yield (see Fig. 4). The goal is to recognize that child characteristics and ASD diagnostic features have implications for MRI and MEG recording, which can be mitigated with appropriately personalized interventions.

For example, children on the autism spectrum (and IDD children) often have co-occurring anxiety and aversion to novel experiences, which impacts their interaction with the MEG machine which is novel, unpredictable, and ungeneralizable. To accommodate the above, systematic desensitization and habituation strategies along with modeling and role-play can be implemented. As such, the MEG visit is personalized for the child, with engagement and reinforcement strategies aligning with the child’s special interests (e.g., watching favorite YouTube videos of elevators (with no sound) during the MEG scan). Clinical/behavioral approaches are integrated with technical advances (e.g., motion compensation) and focus on simple paradigms and obligate responses, culminating in successfully tolerated studies with reproducible results. In our initial study using MEG-PLAN, we demonstrated feasibility in 38 MVNV children with ASD (8 to 12 years old, NVIQ = 46 ± 17) [51]. Scan success for acquirable MEG data occurred at a rate of 76%, and evaluable/analyzable data at a rate of 71% of those acquired, rates consistent with a small set (*N* = 3) of MRI studies in children with significant cognitive impairment [52, 53]. High intra-class correlation coefficient values for M50 latency (*R*^2^ = 0.89) demonstrated reliable measurement of the M50 response even in the presence of significant movement and noise in some participants [51].

MEG-PLAN is conceptually similar to protocols designed for obtaining MRI data in lower-functioning children [52, 53], but provides expanded home-based preparation and is sensitive to research conducted in an often more restrictive medical center environment versus an independent research facility. Combining MEG-PLAN with procedures specific to MRI will allow multimodal imaging studies in MVNV and IDD children. As an example, in our recent work, as MVNV-ASD children showed marked, indeed exacerbated, M50 latency delays [45], it is now of interest to determine whether in low-verbal ASD children such *exacerbated* M50 delays are associated with *more prominent* white matter and/or GABA deficits, and thus whether these measures can be used for stratification across the full ASD population.

## Discussion and future directions

If brain biomarkers have a mechanistic biological basis, such measures may play a role in stratifying the heterogeneous NDD/IDD populations into subpopulations sharing a common biological trait. Such biologically based stratification offers promise in guiding treatment as well as playing a putative role in optimizing clinical trials via selective participant enrichment (e.g., [3]). Such biomarkers may also play a role in revealing an early response (or lack thereof) to a drug (or behavioral) intervention, perhaps allowing for treatment switching, or rational continuance based on evidence of “target engagement” (acute biological response).

In our own research, after identifying atypical auditory encoding in children with ASD without significant language or cognitive impairment, our laboratory worked to better understand these findings via examining auditory encoding processes in other disorders (e.g., disorders that place one at risk for ASD) as well as examining auditory encoding processes in children with significant language or cognitive impairment, including children with IDD. In other studies, via a multimodal approach, we have shown that it is possible to model, or predict, the latency of the auditory M50 response via quantifying the microstructure of auditory pathway white matter (in particular the thalamocortical acoustic radiations). As detailed in section “M50 latency delays in NVIQ-matched IDD children without ASD,” although this approach accounted for more than 50% of the variance in typically developing controls, it was confounded by heterogeneity in a cohort of ~ 100 children with ASD. This heterogeneity, however, allowed identification of a subpopulation of children with ASD whose M50 responses appeared as “outliers” to the TD model (i.e., “unpredictably” long M50s); interestingly, these children showed significantly lower levels of GABA (estimated by advance magnetic resonance spectroscopy) than their ASD peers whose latencies were more consistent with the TD model.

Identification of this group has significant implications for treatment/intervention by identifying a biological basis for stratification (subpopulation definition) and thus a putative biological target for intervention (as well as a means of defining an inclusion criterion for selecting that therapy). It is hoped that future research will extend this work to the scientifically and societally critical group of children with ASD with severe language and cognitive impairments, who are under-included in most imaging research, but whose vital participation is made possible by a combined behavioral and technical protocol we have recently developed, called MEG-PLAN, and its MRI analog MRI-PLAN. Over time, these protocols will enable the use of cutting-edge neuroimaging techniques across the full range of presentations in ASD and IDD, with further personalization of MEG-PLAN and MRI-PLAN, based on identification of factors that maximize scan success, efficiency, and comfort (e.g., temperament profile, cognitive strengths and weaknesses).

Leveraging MEG-PLAN and MRI-PLAN will open up opportunities across the population of children with IDD. As an example, to ascertain the clinical and behavioral implications of a delayed M50 brain response, of interest is examining children with mixed etiology IDD (but not autism), and seeking to identify the relative associations of language impairment, cognitive impairment, and ASD diagnosis to the M50 latency delay, and to investigate the biophysical underpinnings of these associations with multimodal MEG, MRI, and MRS. Such an approach seeks to understand the formidable heterogeneity observed in ASD, NDDs, and IDDs, in general.

A limitation to much of the discussed literature is the general preponderance of males vs females with ASD in these studies, commonly limiting the statistical analysis of sex effects (most studies either recruit exclusively male participants or recruit according to the typical 3–4:1 prevalence, with concomitantly fewer females). Interestingly, by extending the inclusion range with approaches such as MEG-PLAN, it might be possible to access populations (likely more severely impaired individuals) in which canonical male to female prevalences are less markedly different. In any case, future studies are warranted focusing specifically on the role of sex in modulating candidate biomarker quantities.

Additionally, this article concentrates its focus on a particular candidate biomarker, the M50 latency. Several other electrophysiological, and indeed imaging, markers may also offer promise. In our laboratory, for example, we have observed anomalies in the phase synchrony of the auditory gamma-band response; others have noted anomalies in face-processing challenges. The goal of the current paper is not to provide a critical comparative review of all putative biomarkers, but rather to dig deep into the factors that may influence utility, sensitivity, and specificity, for a promising candidate marker (the M50 latency) with much of the logic and philosophy being translatable to other candidate measures.

Finally, and as noted in the “Background” section, in addition to diagnostic/prognostic utility, biomarkers may serve another function—in the design and conduct of pharmaceutical trials via providing an early read-out (after a “test” dose) of the biological effect of a drug. In an acute, dose-escalating trial of a GABA-B agonist, arbaclofen, we found that responsiveness of the M50 latency identified a fraction (*N* = 6) of all the participants (*N* = 25, adolescents with ASD) in whom the drug elicited a significant shortening of M50 latency 1 h post administration [21]. As the response was dose-specific, a role for determining optimal dose is also suggested. Whereas other measures (MRI, MRS) did not clearly identify a subpopulation of “responders,” the individuals defined by their M50 shortening also demonstrated a post-drug increase in their auditory gamma-band phase synchrony, consistent with an acute effect on auditory cortex neural-circuit function and implicating the integrity of the GABAergic system and thus the potential opportunity for a GABAergic drug.

## Conclusion

To conclude, it is our central hypothesis that the latency of the M50 response elicited by simple auditory stimulation may play one or more of several roles as a biological marker in ASD and in neurodevelopment disorders (NDDs) in general. Roles might be in early diagnosis, prognosis given genetic risk, biologically based stratification for clinical trials / treatment, and as indices of early signals of efficacy. Integration of the M50 response in a multimodal characterization of brain structure, neurochemistry, and functional neural activity will augment the specificity of risk characterization in children with developmental delay and intellectual disability. Extension of these studies to infants with genetically identified risk factors might lead to earlier and more nuanced diagnosis/prognosis and, ultimately, earlier intervention. Via the use of advanced imaging and clinical and technical supports (embodied in MEG-PLAN) to personalize the scan experience, such studies are feasible across a broad range of cognitive and language impairments and indeed beginning to offer promise in the pursuit of objective biological markers of brain dysfunction.

## Data Availability

Data will be made available to any scientific investigator upon reasonable request.
